# Working alliance inventory applied to virtual and augmented reality (WAI-VAR): psychometrics and therapeutic outcomes

**DOI:** 10.3389/fpsyg.2015.01531

**Published:** 2015-10-08

**Authors:** Marta Miragall, Rosa M. Baños, Ausiàs Cebolla, Cristina Botella

**Affiliations:** ^1^Department of Personality, Evaluation and Psychological Treatment, University of ValenciaValencia, Spain; ^2^PROMOSAM Excellence in Research Program, Ministry of Economy and CompetitivenessValencia, Spain; ^3^CIBER Fisiopatología Obesidad y Nutrición, Instituto de Salud Carlos IIISantiago de Compostela, Spain; ^4^Department of Basic Psychology, Clinical and Psychobiology, Jaume I UniversityCastellón de la Plana, Spain

**Keywords:** alliance, virtual reality, augmented reality, psychometrics, clinically significant change

## Abstract

This study examines the psychometric properties of the Working Alliance Inventory-Short (WAI-S) adaptation to Virtual Reality (VR) and Augmented Reality (AR) therapies (WAI-VAR). The relationship between the therapeutic alliance (TA) with VR and AR and clinically significant change (CSC) is also explored. Seventy-five patients took part in this study (74.7% women, *M*_age_ = 34.41). Fear of flying and adjustment disorder patients received VR therapy, and cockroach phobia patients received AR therapy. Psychometric properties, CSC, one-way ANOVA, Spearman’s Correlations and Multiple Regression were calculated. The WAI-VAR showed a unidimensional structure, high internal consistency and adequate convergent validity. “Not changed” patients scored lower on the WAI-VAR than “improved” and “recovered” patients. Correlation between the WAI-VAR and CSC was moderate. The best fitting model for predicting CSC was a linear combination of the TA with therapist (WAI-S) and the TA with VR and AR (WAI-VAR), due to the latter variable slightly increased the percentage of variability accounted for in CSC. The WAI-VAR is the first validated instrument to measure the TA with VR and AR in research and clinical practice. This study reveals the importance of the quality of the TA with technologies in achieving positive outcomes in the therapy.

## Introduction

*Therapeutic alliance* has been broadly defined as the cooperation between the patient and therapist in their therapeutic work (Bachelor and Horvath, 1999). The most distinguishing feature of the current alliance’s conceptualization is the emphasis on collaboration and consensus (Bordin, 1980; Hatcher et al., 1995). For instance, Greenson (1965) defines the alliance as a reality-based collaboration between patient and therapist, and Luborsky’s (1976) model highlights one type of alliance that represents the collaborative relationship between patient and therapist to overcome the patient’s problems. It may be defined as the patient experience of therapy as a process of working together to achieve goals and it is experienced more typically in the later phases of the therapy. However, Bordin is one of the most influential authors in the study of this concept. Bordin (1979) named TA as *working alliance*, and conceptualized it as a pan-theoretical concept that is applicable to any therapeutic approach (Horvath and Luborsky, 1993). It consists of three processes in therapy: (a) agreement about the therapeutic goals; (b) consensus on the tasks included in the therapy; and (c) bonds between patient and therapist (Bordin, 1979).

Evidence has shown that TA is one of the main ingredients in any type of therapy (Horvath and Symonds, 1991; Hubble et al., 1999; Martin et al., 2000). Indeed, TA has been postulated as a predictor of improvement in therapy, global scores of satisfaction and dropouts (Bordin, 1979), and TA quality has been considered even more important than the type of treatment in predicting positive therapeutic outcomes (Safran and Muran, 1995). The meta-analysis conducted by Martin et al. (2000) showed that the overall relationship between TA and therapeutic outcomes was moderate, but robust (accounting for approximately 5% of the variance in therapeutic outcomes). Furthermore, the relationship between TA and therapeutic outcomes did not appear to be influenced by other moderator variables, such as the outcome measure used in the study, the outcome rater, the time of alliance assessment, the alliance rater, the type of treatment provided, or the publication status of the study. More recently, Horvath et al. (2011) also found that the overall relation between TA and therapeutic outcomes in individual psychotherapy was robust, accounting for 7.5% of the variance in therapeutic outcomes, independently of other variables. This moderate but strong relationship between TA and therapeutic outcomes across a broad spectrum of treatments in a variety of patients and problem contexts has been a consistent finding in different studies (Horvath and Symonds, 1991; Horvath and Bedi, 2002).

Many measures have been developed to assess TA, but there is a lack of agreement on a unifying alliance model and a set of measures. The meta-analysis conducted by Horvath et al. (2011) showed that over 30 alliance measures were used in 201 different studies, such as *California Psychotherapy Alliance Scales* (CALPAS, Gaston and Marmar, 1994), *Helping Alliance Questionnaire* (HAQ, Alexander and Luborsky, 1987), *Vanderbilt Psychotherapy Process Scales* (VPPS, O’Malley et al., 1983), and *Working Alliance Inventory* (WAI, Horvath and Greenberg, 1986, 1989). However, the most widely used questionnaire is the *Working Alliance Inventory* (WAI) (Martin et al., 2000; Horvath et al., 2011), which was developed to measure the working alliance as defined by Bordin (1979) in all types of therapy. The WAI is also available in a short version (WAI-S, Tracey and Kokotovic, 1989), both for patients and therapists.

Although a large amount of literature has been published about TA in traditional face-to-face therapeutic contexts (Horvath et al., 2011), TA research on therapy supported by ICTs has been neglected (Emmelkamp, 2005). ICT-supported therapies have been used for more than a decade (Barak et al., 2008), but a recent systematic review of TA in e-therapy showed the low number of studies in this field (Sucala et al., 2012). In this review, the definition of e-therapy by Manhal-Baugus (2001) was used: “a licensed mental health care professional providing mental health services via e-mail, video conferencing, VR technology, chat technology, or any combination of these.”

The most frequently used ICTs in the field of psychological treatments are the Internet, mobile phones, PDAs, PCs, VR, and AR (Botella et al., 2007a). The present study focuses on therapies supported by VR and AR, whose use has experienced a strong growth in psychological treatments in recent years (Quero et al., 2012). The results of a meta-analysis showed that VR exposure therapy is highly effective in treating phobias (Powers and Emmelkamp, 2008), and some studies have already shown encouraging preliminary efficacy data on the treatment of more complex disorders such as Panic Disorder and Agoraphobia, Posttraumatic Stress Disorder, and Eating Disorders (Botella et al., 2007b). Moreover, empirical studies have also demonstrated the efficacy of AR in the treatment of specific small animal phobias (spiders and cockroaches) and acrophobia (Botella et al., 2009).

Even though empirical studies have shown the clinical effectiveness of therapies supported by VR and AR for several conditions, mental health professionals have some concerns about the use of these technologies in clinical practice, such as those remarked by Meyerbröker and Emmelkamp (2008) about the effect of the specific devices of VR (e.g., head-mounted device) over the relationship between the patient and the therapist. However, several studies do not find support for these concerns. Regarding AR, Wrzesien et al. (2013) compared the development of TA in individuals with small animal phobia who were treated with AR exposure therapy and *in vivo* exposure therapy, and no differences were found between the two conditions. Therefore, these authors concluded that AR did not have a negative influence on TA. Regarding VR, Ngai et al. (2015) compared TA in patients with social anxiety disorders who were treated with VR exposure therapy and *in vivo* exposure group therapies. Contrary to their initial hypothesis, they did not find lower TA in the VR group. However, research on the role of TA during ICT-supported therapies is scarce, and there is a clear need for further studies investigating the underlying process in these treatments (Meyerbröker and Emmelkamp, 2010).

Focusing specifically on VR therapy and therapeutic outcomes, Meyerbröker and Emmelkamp (2008) studied TA in VR exposure therapy for specific phobias (fear of flying and acrophobia), and they concluded that the quality of TA predicted successful therapeutic outcomes in terms of anxiety reduction for the fear of flying group. In a more recent work, these authors also found positive moderate correlations between TA and therapeutic outcomes in patients with panic disorder and agoraphobia treated with VR therapy (Meyerbröker et al., 2013).

Although these preliminary studies are encouraging, more research is needed to understand TA in these therapies (Meyerbröker and Emmelkamp, 2008; Sucala et al., 2012). In this sense, Elvins and Green (2008) argued that TA measures have been challenged by the current variety of therapeutic interventions that go beyond the classic interpersonal encounter (such as non-interpersonal computer-aided interventions). TA is still conceptually in its psychotherapeutic roots, and it is necessary to test more generic concepts. Thus, these authors have suggested that one future task should be to further examine the conceptual underpinnings of TA through the use of experimental designs to identify the most valid conceptualization, refine its measurement, and make it more specific. Therefore, this study aims to adapt the WAI-S scale to therapies supported by AR and VR. We will use the short version of the WAI because the results of its validation showed that the factor structure was similar to the original WAI, and the reduction of items increases its usefulness in terms of administration (Tracey and Kokotovic, 1989). To our knowledge, there are no questionnaires that explore TA with ICTs, and it is relevant to have an adequate instrument with appropriate psychometric properties that allows researchers and clinicians to assess TA in ICT-supported therapies. Furthermore, research in this field will help to address concerns about TA in ICT-supported therapies and its relation to therapeutic outcomes. In addition, no studies have explored the relationship between TA with ICTs and therapeutic outcomes in terms of CSC. In this sense, it is extremely important for clinical practice to have an instrument that helps to predict the therapeutic outcomes of patients in ICT-supported therapies.

The aims of this study are: (1) to examine the psychometric properties of the WAI-S adaptation to VR and AR (WAI-VAR); (2) to explore the relationship between the WAI-S (patient-therapist) and WAI-VAR (patient-VAR); and (3) to analyze the relationship between the WAI-S, WAI-VAR and CSC.

## Materials and Methods

### Participants

Seventy-five Spanish participants took part in this study (*M* = 34.41 years old; *SD* = 10.48; 74.7% women and 25.3% men). All of them were Caucasian. Patients were recruited and treated in the Psychological Support Service of Jaume I University (Spain) by seven psychologists with similar clinical experience. All participants were informed about the study and signed the informed consent documents before beginning the therapy in accordance with the Declaration of Helsinki. Moreover, the study was approved by the Internal Review Board at Jaume I University. Each patient was assigned to a psychologist based on his or her time availability. Participants were interviewed and received a primary diagnosis of cockroach phobia (*n* = 40), fear of flying (*n* = 20) or adjustment disorder (*n* = 15), according to the *Diagnostic and Statistical Manual of Mental Disorders-Text Revised* (DSM-IV-TR; American Psychiatric Association [APA], 2000). None of them had any comorbidities. Participants received the following treatments (see **Table 1**).

**Table 1:** Clinical characteristics of the sample and therapies.

Primary diagnosis	*N*	Kind of ICT	Number of sessions
Cockroach phobia	40	AR	One session of 3 intensive hours
Fear of flying	20	VR	Six sessions (3 weeks)
Adjustment disorder	15	VR	Six sessions (6 weeks)

*N = Number of patients with each primary diagnosis; ICT = Information and Communication Technology.*

#### Fear of Flying Patients Received Six Sessions (Over 3 Weeks) of VR Exposure Therapy

The main component of this therapy was VR exposure, but patients also received educational information about anxiety, flying, exposure, and advantages of VR in the first session. The objective of the following six sessions was the exposure of the patient to three virtual scenarios: (1) packing at home, (2) waiting for boarding at the airport, and (3) sitting in the airplane while taking off and during flight. The VR exposure progressed from the less to the most anxious situations (according to the hierarchy established in the first session). An exhaustive description of the treatment can be found in Botella et al. (2004).

#### Adjustment Disorder Patients Received Six Weekly Sessions of VR

We used “EMMA’s World,” a virtual environment used to enhance the emotional experience in which patients could explore their negative experiences for their specific therapeutic needs. The system showed customized and clinically significant environments (e.g., different landscapes associated with different emotions, photos, phrases, etc.) for each patient where they could feel free to express their emotions and thoughts. The objective was to obtain a physical representation of the personal meanings and emotions that were related to the patient’s negative experience in order to activate, structure or restructure the negative experiences. The treatment consisted of 6 weekly sessions (one for the “educational component,” four for “exposure,” and one for “relapse prevention”). For a detailed description see Baños et al. (2009).

#### Cockroach Phobia Participants Received an Intensive 3-h Session of AR Exposure Therapy

The main objective of this therapy was the exposure of the patient to the virtual cockroach in the real environment. The system included options that enabled the therapist to apply the treatment progressively: number of cockroaches, movement of cockroaches (static or moving), size of cockroaches (small, medium, or large), and the possibility to “kill” cockroaches. The therapy was applied using the guidelines of “one-session treatment” recommended by Öst et al. (1991), which implied utilizing intensive exposure, and it was carried out in only one session in a maximum of 3 h. The exposure exercises were defined during the diagnostic interview, hierarchically organized from the least to the most anxious situation, and each exposure exercise was first modeling by the therapist. An extensive description of the treatment can be found in Botella et al. (2005).

### Measures

The following questionnaires were used to evaluate TA with the therapist and TA with VR and AR:

#### Working Alliance Inventory – Short Version (WAI-S, Tracey and Kokotovic, 1989; Spanish Adaptation by Corbella et al., 2011)

The WAI-S is the short version of the WAI (Horvath and Greenberg, 1986), where each subscale represents Bordin’s multidimensional theoretical conceptualization of TA (Goals, Tasks, and Bonds). The WAI-S consists of 12 items, and each item is rated on a 7-point Likert scale (1 = never; 7 = always). Each subscale is assessed with four items: (a) Goals (items 4, 6, 10, 11): the extent to which patient and therapist agree on the overall treatment goals. The patient is aware that these goals are relevant and identifies with the subjects made explicit and implicit during the therapy; (b) Tasks (items 1, 2, 8, 12): the extent to which client and therapist agree on the tasks that are relevant for achieving these goals. The patient feels that the tasks agreed upon during the therapy are rational, reachable and related to the therapeutic goals; and (c) Bonds (items 3, 5, 7, 9): the extent of emotional bonding between patient and therapist in terms of trust and attachment. Some of the facilitative conditions that help to foster this bond are mutual understanding, a caring attitude by the therapist, and the patient’s perception that the therapist likes him or her. The questionnaire provides four scores: three subscale scores and an aggregate overall score. It has two inverted items (items 4 and 10). The total score ranges from 12 to 84, with higher scores reflecting a stronger working alliance. The mean and standard deviation of this sample were *M* = 73.31 and *SD* = 9.29.

#### Working Alliance Inventory applied to VR and AR (WAI-VAR)

This is an adaptation of the WAI-S elaborated by the authors. It includes 12-items to be answered on a 7-point Likert rating scale (1 = never; 7 = always) to yield a total aggregate score for alliance quality and three subscale scores (Goals, Tasks, and Bonds). The WAI-VAR consists of the same subscales and provides the same scores as the WAI-S. For this sample, *M* = 65.93 and *SD* = 12.74. Details about its adaptation appear in the Section “Procedure.”

The following questionnaires were used during pre-test and post-test in order to assess the CSC. These scores were used to calculate the Reliable Change Index (Jacobson and Truax, 1991) to find out how much change occurred at the end of the treatment.

#### Fear of Cockroach Questionnaire (FCQ; Nebot et al., 2012; Spanish Cockroach Adaptation of Fear of Spiders Questionnaire; FSQ; Szymanski and O’Donohue, 1995)

The FCQ is an 18-item self-report questionnaire assessing cockroach phobia. Participants rate their agreement with statements such as “Cockroaches are one of my worst fears” on a 7-point Likert type scale (0 = strongly disagree; 7 = strongly agree). The total score ranges from 0 to 108, with a cut-off of 15 or more reflecting at least a midlevel fear of cockroaches. The mean and standard deviation for this sample were *M* = 95.93 and *SD* = 15.77. This version of the questionnaire showed high internal consistency in the Nebot et al. (2012) validation in their clinical sample (α = 0.86), and two factors were found (“avoidance and help-seeking” α = 0.86; and “surveillance and fear of harm” α = 0.62).

#### Fear of Flying Scale (FFS; Haug et al., 1987)

This is a 21-item self-report scale on which the participant rates his or her level of anxiety in different flying-related situations (scale ranging from 1 to 4). It consists of three subscales assessing (a) flying-related anxiety situations; (b) typical moments before the flight; and (c) typical moments during the flight. The total score ranges from 21 to 84. The original version of the questionnaire showed high internal consistency in the clinical sample (α = 0.94). In this study, the Spanish translated version by Bornas et al. (2012) was used. Cronbach’s alpha and 15-day re-test reliability were α = 0.95 and α = 0.86, respectively, in a similar sample (unpublished results). The mean and standard deviation for this sample were *M* = 62.88 and *SD* = 7.19.

#### Positive and Negative Affect Scales (PANAS; Watson et al., 1988; Spanish Adaptation by Sandín et al., 1999)

The PANAS consists of two 10-item mood scales, and was developed to provide brief measures of positive and negative affect. Respondents are asked to rate the extent to which they have experienced each particular emotion within a specified time period, using a 5-point scale. The scale points are: 1 = very slightly or not at all; 2 = a little; 3 = moderately; 4 = quite a bit; 5 = very much. The scales were shown to be highly internally consistent, largely uncorrelated, and stable at appropriate levels over a 2-month time period in the original validation. We only used the Negative Affect Scale in the “moment” (not in general), which ranged from 5 to 50. For the Spanish translated version by Sandín et al. (1999), Cronbach’s alpha of the Negative Affect Scale was α = 0.91 for men and α = 0.89 for women. The mean and standard deviation for this sample were *M* = 25.20 and *SD* = 5.88.

### Procedure

Following the recommendations by Hambleton and Patsula (1999), we carried out an adaptation of the patient version of the WAI-S (Corbella et al., 2011) in order to evaluate the relationship between TA and VR and AR, as defined by Bordin (1979). Thus, the purpose of the WAI-VAR is to measure agreement about goals and tasks between the patient and the “virtual environment,” and the comfort-trust in the virtual environment. Therefore, we replaced the words “my therapist” or “therapy” with “virtual environment.” For instance, item 2 (“What I am doing in therapy gives me new ways of looking at my problem) was transformed in this way: “What I am doing in the virtual environment gives me new ways of looking at my problem” (see **Table 2**).

**Table 2:** Psychometric properties of the WAI-VAR: Skewness and Kurtosis Index, Mean (*M*), and Standard Deviation (*SD*), factorial loadings (*λ*) with a one-factor structure using Maximum Likelihood (ML) and communalities (*h*^2^).

	Skewness index	Kurtosis index	*M* (*SD*)	λ	*h*^2^
Item 1. The virtual environment helps me to improve my situation.	–0.62	-0.56	5.45 (1.39)	0.84	0.70
Item 2. What I am doing in the virtual environment gives me new ways of looking at my problem.	–1.46	2.83	5.80 (1.28)	0.85	0.72
Item 3. I feel comfortable in the virtual environment.	–1.21	2.03	5.60 (1.28)	0.60	0.36
Item 4. What I am doing in the virtual environment does not help me accomplish what I want to achieve in therapy.	–0.59	-1.19	4.73 (2.16)	0.37	0.14
Item 5. I trust in the virtual environment’s ability to help me.	–1.50	2.96	5.81 (1.28)	0.79	0.63
Item 6. The virtual environment is sensitive to the therapeutic goals that my therapist and I have agreed on.	–1.20	1.26	5.40 (1.39)	0.56	0.32
Item 7. I feel received by the virtual environment.	–1.02	1.26	5.29 (1.27)	0.78	0.60
Item 8. The virtual environment works on the important things that I think I should work on in therapy.	–1.28	2.50	5.84 (1.19)	0.82	0.67
Item 9. I trust in the virtual environment.	–1.03	0.64	5.67 (1.31)	0.86	0.74
Item 10. The virtual environment does not work on the important problems that it should.	–0.73	-1.15	5.03 (2.28)	0.33	0.11
Item 11. Thanks to the virtual environment I have achieved a good understanding of the kind of changes that would be good for me.	–1.07	0.93	5.59 (1.49)	0.86	0.74
Item 12. The way the virtual environment works on my problems is correct.	–1.21	1.33	5.72 (1.39)	0.89	0.79

Once the WAI-VAR had been adapted, patients receiving VR therapy completed the WAI-S (Corbella et al., 2011) and the WAI-VAR at the end of the third psychotherapy session, while patients receiving AR therapy completed both questionnaires after an intensive 3-h therapeutic session. The patients also filled out other questionnaires before the therapy (pre-test). Cockroach phobia patients completed the FCQ (Spanish cockroach adaptation of the FSQ, Szymanski and O’Donohue, 1995), fear of flying patients completed the FFS (Haug et al., 1987), and adjustment disorder patients completed the PANAS (Watson et al., 1988; Sandín et al., 1999). At the end of the therapy, patients completed the same questionnaires (post-test).

### Data Analysis

The statistical analyses were conducted using the Statistical Package for the Social Sciences (SPSS) for Windows, version 19. Before starting the statistical analyses, missing item values were analyzed and imputed using the Expectation–Maximization Algorithm method (Schafer, 1997). Several statistical procedures were performed. Descriptive statistics (skewness and kurtosis) were used to check the normality of the data (Fabrigar et al., 1999). The suitability of the data for Exploratory Factor Analysis (EFA) was assessed using the Kaiser-Meyer-Olkin (KMO) and Barlett’s Test of Sphericity. To determine the number of factors retained on the EFA, Parallel Analysis (Horn, 1965) was applied using a macro for SPSS (O’Connor, 2000). In addition, the Standardized Root Mean Square Residual (SRMR) was calculated manually, as some authors recommend, complementing Parallel Analysis with analysis of residuals (Abad et al., 2011). To explore the factor structure of the WAI-VAR, an EFA was conducted using a Maximum Likelihood (ML) estimation extraction method. ML was chosen because the data were relatively normally distributed (Fabrigar et al., 1999).

Internal consistency of the WAI-VAR subscales and total score was assessed using Cronbach’s alpha coefficient (Cronbach, 1951). Convergent validity was also assessed using correlation coefficient analysis between the WAI-S and WAI-VAR.

In addition, pre-test and post-test scores were examined for each patient, and the Reliable Change Index (RCI) was calculated to examine the CSC (Jacobson and Truax, 1991). Therefore, based on Jacobson and Truax (1991), as we did not have data from the general population or a functional population, we chose “a” criterion to decide when a patient had achieved a clinically significant improvement: the post-test score had to be 2 *SD* in the direction of functionality above the mean for a dysfunctional population, that is, *M*_dysfunctional_ ± 2 *SD*. Then we calculated the RCI to analyze the second condition to test the CSC, where an RCI equal to or greater than | 1.96| (*p* < 0.05) indicates a reliable change. To calculate the RCI, we used the post-test mean (*X*_post_) and the pre-test mean (*X*_pre_) of the result achieved for each patient, and the mean dysfunctional *(M*_dys_), the standard deviation (*SD*) and the stability reliability (*r*_xx_) of the Muris and Merckelbach (1996) FSQ validation, Haug et al. (1987) FFS validation and Watson et al. (1988) PANAS validation. Finally, taking both criteria into account, participants were classified into four categories: (a) *Recovered*. When the change is significantly reliable (RCI ≥ | 1.96| ; *p* < 0.05) and the post-treatment score is located within the range of the functional distribution (*M* ± 2 *SD*); (b) *Improved.* When the change is significantly reliable (RCI ≥ | 1.96| ; *p* < 0.05), but the post-treatment score does not reach the functional level; (c) *Not changed.* When the change is not significantly reliable and the post-treatment score does not reach the functional level; (d) *Deteriorated.* When the change is significantly reliable (RCI ≥ | 1.96| ; *p* < 0.05), but the post-treatment score is worse than the pre-treatment score. Moreover, in order to find out whether there were significant differences in the TA among these participant categories, a one-way ANOVA was carried out. When a significant overall group difference was found, *post hoc* tests were performed to determine which group comparisons were significant, using a Bonferroni adjustment.

Finally, Spearman’s correlations and multiple regression analyses (using the hierarchical entering method) were calculated to examine the relationship between the WAI-VAR and CSC, and the relationship between the WAI-S and CSC.

## Results

### Psychometric Properties of WAI-VAR

#### Exploratory Factor Analysis

First, the percentage of missing values was explored, finding a random missing value percentage ranging from 0 to 2.7% per item. Items’ missing values were imputed using the Expectation–Maximization Algorithm method (Schafer, 1997). Then, the sample’s normality was analyzed, assuming the multivariate normality of the variables, as skewness values were <| 2|, and kurtosis values were <| 7| (West et al., 1995; Russell, 2002) (see **Table 2**), ML extraction was selected (Fabrigar et al., 1999). Both the KMO value (0.89) and the Barlett’s Test of Sphericity value [*x*^2^(66) = 645.644, *p* < 0.001] revealed that it was appropriate to perform a factor analysis. Regarding the number of factors to extract, Parallel Analysis (Horn, 1965) showed that one factor had to be retained because only one factor had an eigenvalue (raw data eigenvalue = 6.87) greater than the eigenvalue at the 95th percentile for randomly generated data (percentile 95th eigenvalue = 1.89) (Fabrigar and Wegener, 2012). In addition, we determined that the unidimensional model fit the data correctly because SRMR <0.08 (SRMR = 0.076) (Hu and Bentler, 1999). Then, factorial rotation with one factor was carried out using the ML extraction method, which showed that one factor explains 54.21% of the total variance. All the item communalities had values above 0.30, except item 4 (*h*^2^ = 0.14) and item 10 (*h*^2^ = 0.11). The factorial solution showed that all the items had minimum factor loadings of 0.30 (see **Table 2**).

#### Reliability Analysis: Internal Consistency

Cronbach’s alpha coefficient for the WAI-VAR was high for the overall scale (α = 0.906). We analyzed the item-total correlation, and by excluding items 4 and 10, the alpha value for the overall scale increased slightly (α = 0.913 and α = 0.919, respectively). The alpha values for the subscales were 0.70 for “Goals,” 0.92 for “Tasks,” and 0.86 for “Bonds,” although the latter results were not as relevant due to the unidimensionality of the AFE results.

#### Convergent Validity: Correlation with WAI-S

The relationship between the WAI-VAR and WAI-S was examined. Pearson’s correlation showed a large correlation between these two total scores (*r* = 0.70, *p* < 0.001). Moreover, Pearson’s correlations were calculated for each pair of items on the two questionnaires: item 1 (*r* = 0.41, *p* < 0.001), item 2 (*r* = 0.65, *p* < 0.001), item 3 (*r* = 0.27, *p* = 0.021), item 4 (*r* = 0.57, *p* < 0.001), item 5 (*r* = 0.39, *p* < 0.001), item 6 (*r* = 0.19, *p* = 0.113), item 7 (*r* = 0.42, *p* < 0.001), item 8 (*r* = 0.31, *p* < 0.001), item 9 (*r* = 0.60, *p* < 0.001), item 10 (*r* = 0.65, *p* < 0.001), item 11 (*r* = 0.38, *p* < 0.001), and item 12 (*r* = 0.60, *p* < 0.001). All the items had significant correlations, except item 6 (“My therapist and I are working toward mutually agreed upon goals” and “The virtual environment is sensitive to the therapeutic goals that my therapist and I have agreed on.”).

### The Relationship between WAI-VAR and CSC

To determine the CSC (Jacobson and Truax, 1991), all participants were classified into three categories, taking into account the post-treatment score and the RCI score. Forty percent of the patients (*n* = 30) were “recovered,” 26.7% (*n* = 20) were “improved,” and 33.3% (*n* = 25) were “not changed.”

In order to find out whether there were significant differences between these participant categories on the WAI-VAR, a one-way ANOVA was carried out. A significant effect of group, *F*(2,72) = 17.25, *p* < 0.001, $\eta_{p}^{2}$ = 0.32, was found. According to Cohen’s (1988) indications, the effect size was large ($\eta_{p}^{2}$ > 0.14). *Post hoc* comparisons using the Bonferroni correction revealed that the mean score for the “not changed” patients (*M* = 56.12; *SD* = 11.57) was significantly different from the “recovered” (*M* = 72.77; *SD* = 8.64), *p* < 0.001, and “improved” patients (*M* = 67.95; *SD* = 12.01), *p* < 0.001. However, there were no significant differences (*p* = 0.362) between “recovered” and “improved” patients on the WAI-VAR scores.

**FIGURE 1:**
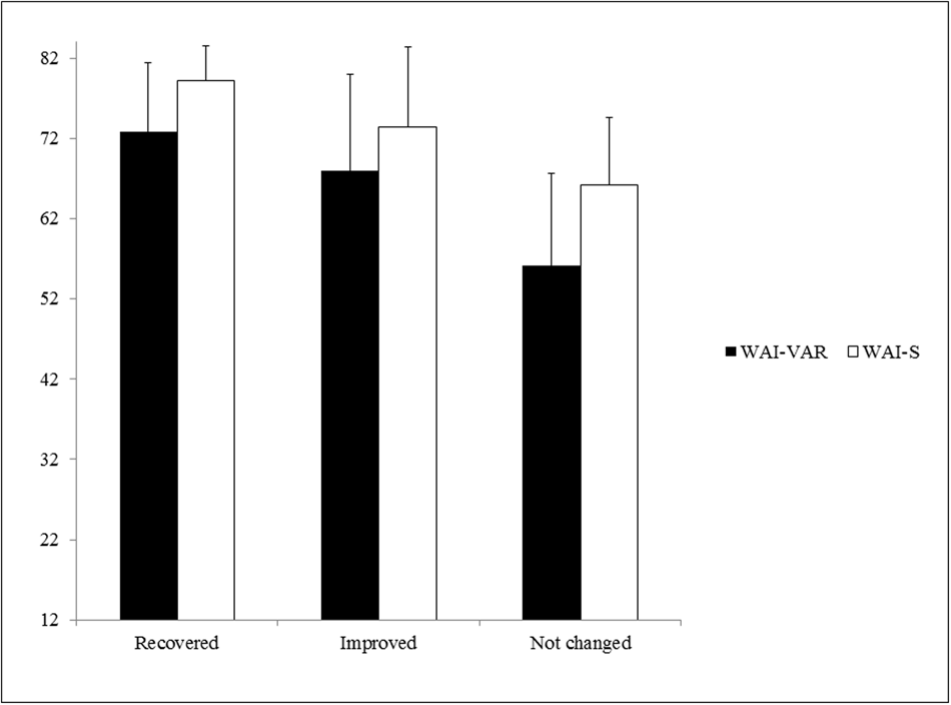
**Results on the WAI-VAR and WAI-S of the “recovered,” “improved,” and “not changed” patients.** Mean (*M*) and Standard Deviation (*SD*) on the WAI-VAR and WAI-S of the “recovered,” “improved,” and “not changed” patients. WAI-S = Mean and Standard Deviation on the Working Alliance Inventory-Short Form of the “recovered,” “improved,” and “not changed” patients; WAI-VAR = Mean and Standard Deviation on the Working Alliance Inventory applied to Virtual and Augmented Reality of the “recovered,” “improved,” and “not changed” patients.

Furthermore, a significant effect of group was also found for the WAI-S scores, *F*(2,72) = 19.98, *p* < 0.001, $\eta_{p}^{2}$ = 0.36. According to Cohen’s (1988) indications, the effect size was also large ($\eta_{p}^{2}$ > 0.14). *Post hoc* comparisons using the Bonferroni correction indicated that the mean score for the “recovered” patients (*M* = 79.17; *SD* = 4.35) was significantly different from the “improved” (*M* = 73.36; *SD* = 10.01), *p* = 0.029, and “not changed” (*M* = 66.24; *SD* = 8.30), *p* < 0.001, patients’ scores (see **Figure 1**).

Spearman’s correlation (*r*_s_) between the WAI-VAR and CSC showed a significant relationship (*r*_s_ = 0.55, *p* < 0.001). Moreover, the relationship between the WAI-S and CSC was significant (*r*_s_ = 0.61, *p* < 0.001). Thus, according to Cohen (1988), the relationships were large (*r >* 0.50) in both cases. In addition, the effect size was calculated with the Determination Coefficient (*R*^2^)_,_ showing that the WAI-VAR and CSC shared 30% (*R*^2^ = 0.30) of the variance, and the WAI-S and CSC shared 37% (*R*^2^ = 0.37) of the variance.

Finally, multiple regression analyses (using the hierarchical entering method) was performed to investigate the WAI-VAR in order to predict the level of CSC after controlling for the WAI-S. Preliminary analyses were conducted to ensure that the assumptions of normality, linearity, and homoscedasticity were not violated (Tabachnick and Fidell, 2007). In the first step of the multiple regression, the WAI-S was entered. This model was statistically significant, *F*(1,73) = 40.32, *p* < 0.001, and explained 36% of the variance in CSC. After entering the WAI-VAR in the second step, the total variance explained by the model as a whole was 39%, *F*(2,72) = 23.40, *p* < 0.001. The introduction of the WAI-VAR explained an additional 3% variance in CSC, after controlling for the WAI-S, *R*^2^ = 0.03; *F*(1,72) = 4.54; *p* = 0.037. In the final model, the two predictors were statistically significant, with the WAI-S recording a higher Beta value (β = 0.60, *t* = 6.35, *p* < 0.001) than the WAI-VAR (β = 0.27, *t* = 2.13, *p* = 0.037) (see **Table 3**). Results of multiple regression indicated that the best fitting model for predicting CSC was a linear combination of the WAI-S and the WAI-VAR because the WAI-VAR slightly increased the percentage of variability accounted for in the CSC from 36 to 39%.

**Table 3:** Multiple regression of CSC.

CSC							
**Predictors**	***R***	***R*^2^**	***R*^2^ change**	***B***	***SE***	**β**	***t***

**Step 1**							
Constant				-0.30	0.64		-4.64^∗∗∗^
WAI-S	0.60	0.36^∗∗∗^		0.06	0.01	0.60^∗∗∗^	6.35^∗∗∗^
**Step 2**							
Constant				-2.90	0.63		-4.62^∗∗∗^
WAI-S				0.04	0.01	0.41^∗∗^	3.18^∗∗^
WAI-VAR	0.63	0.39^∗^	0.03^∗^	0.02	0.01	0.27^∗^	2.13^∗^

*Statistical significance: ^∗^p < 0.05; ^∗∗^p < 0.01; ^∗∗∗^p < 0.001. CSC, Clinically Significant Change; WAI-S, Working Alliance Inventory-Short Form; WAI-VAR, Working Alliance Inventory with Information and Communication Technologies. R, Multiple Correlation Coefficient; R^2^, Coefficient of determination; R^2^ Change, Coefficient of determination Change; B, Unstandardized coefficient; SE, Standard Error; β, Beta coefficient; t, t-statistic (estimated coefficient divided by its own SE).*

## Discussion

The aims of this study were to explore the psychometric properties of the WAI-S adaptation to ICTs (WAI-VAR) in therapies supported by VR and AR, explore the relationship between the WAI-S and WAI-VAR, and observe the relationship between the WAI-S, WAI-VAR, and CSC. The WAI-VAR is the first questionnaire to measure the TA with ICTs, and this is the first study to examine the relationship between the TA with ICTs and CSC.

Results on the EFA of the WAI-VAR showed a unidimensional structure, which is not in line with the three-dimensional Bordin’s (1979) theory of TA or the hierarchical bi-level model proposed by the authors of the WAI-S (Tracey and Kokotovic, 1989). However, other authors, such as Corbella et al. (2011), also found a unidimensional structure. Corbella et al. (2011) administered the Spanish version of the WAI-S to a sample of 229 patients receiving psychotherapy when they finished the third session, and they found that the internal consistency reliability was good for the total score (α = 0.91) and subscales (Goals α = 0.85; Tasks α = 0.88; and Bonds α = 0.86). However, factor analysis did not fit well with the structure that would be expected from Bordin’s (1979) theory of TA or the structure proposed by Tracey and Kokotovic (1989) because all the items except two (item 4 and item 10) loaded on the first factor. Therefore, Corbella et al. (2011) concluded that the WAI-S would have one factor or two highly correlated factors. In this sense, we can conclude that the WAI-VAR is more unidimensional in practice, even though we can differentiate its dimensions in theory.

Regarding the items, it should be highlighted that inverted items had communalities values under *h*^2^ = 0.30 (item 4 and item 10), and they had the lowest factor loadings on the questionnaire. These items were also problematic in Corbella et al.’s (2011) validation. Thus, it seems useful to rewrite these items in a positive sense. In order to refine the adaptation, better differentiate the three dimensions, and solve the problem with the inverted items, we propose changes in item 1 (“The virtual environment focuses on the things I have to do to improve my situation”), item 4 (“What I am doing in the virtual environment helps me to accomplish what I want to achieve in therapy”), and item 10 (“The virtual environment and I have the same ideas about what my problems are”).

Regarding reliability, the internal consistency value for the overall scale was good, but lower than the one reported by Corbella et al. (2011). However, when items 4 and 10 were excluded, the alpha value increased slightly (α = 0.913 and α = 0.919, respectively). This result can be explained by a poor comprehension of double negatives in the Spanish population. Hence, this result reaffirms the need to rewrite these items.

Regarding the convergent validity with the WAI-S, a large correlation between the two measures was found, as well as significant correlations between each pair of items on the two questionnaires, except item 6 (“My therapist and I are working towards mutually agreed upon goals” and “The virtual environment is sensitive to the therapeutic goals that my therapist and I have agreed on.”). This result could indicate that the adaptation of this item was appropriate, as it seems difficult to agree about anything with a computer. We propose changing the word “sensitive” to “works to obtain” (“The virtual environment works to obtain the therapeutic goals that my therapist and I have agreed on”).

Regarding the relationship between the WAI-VAR and CSC, there were significant differences in the scores among “recovered,” “improved,” and “not changed” patients. Specifically, “not changed” patients scored lower than “improved” and “recovered” patients. Similar results were also found for the WAI-S. This result shows that the “not changed” patients did not achieve as high a TA as “improved” or “recovered” patients.

In addition, significant large relationships were found between the WAI-VAR and CSC, and between the WAI-S and CSC. These results are in line with previous meta-analytical results (Horvath and Symonds, 1991; Horvath and Bedi, 2002; Horvath et al., 2011). It must be highlighted that it is the first time that a relationship between the TA with ICTs and CSC has been found in therapies supported by VR and AR, showing similar explained variance to the TA with a therapist.

Regarding the contribution of the WAI-VAR and WAI-S to predicting the CSC, results of multiple regression analyses showed that adding the WAI-VAR to the model slightly increased the percentage of variability accounted for in the CSC. In other words, the WAI-S explains 36% of the CSC, but if the WAI-VAR contribution is added, the model explains 39% of the CSC. Hence, our results suggest that the quality of the TA, both with ICTs and with a therapist, is a very important aspect of VR- and AR-supported therapies in achieving a CSC in the patients.

These results suggest that concerns about the difficulty of creating the TA between the patient and therapist using ICTs (Wrzesien et al., 2013; Ngai et al., 2015) are not justified. The participants in this study scored high on the WAI-S, in spite of the use of VR and AR in the therapy. These results coincide with conclusions reported by Meyerbröker and Emmelkamp (2008), Meyerbröker et al. (2013), Wrzesien et al. (2013), and Ngai et al. (2015). Furthermore, it is extremely important to pay attention to the quality of the WAI-VAR, because it increases positive therapeutic outcomes.

Limitations of the current study should be noted. First, the size of the sample should be larger to provide greater support for the EFA. Second, the sample is composed of participants suffering from three different mental disorders and receiving treatments using two different ICTs (VR and AR). Finally, the TA was measured at different times (in the third session for VR and in the first intensive three-hour session for AR). However, the number of hours of therapy was equivalent, as all the patients had received three hours of therapy when they completed the questionnaire. In addition, some studies indicate that the extent of TA is established in the first sessions, regardless of the number of sessions (Norcross, 2011).

In any case, future studies should administer this questionnaire in a larger sample with refined items (see Appendix 1), in the same time (session) and using the same ICT. Moreover, future studies should carry out a confirmatory factor analysis with a larger sample in order to verify the dimensionality of the WAI-VAR. In addition, in order to determine the causality of TA on predicting CSC, it would be interesting to explore whether the WAI-VAR predicts symptoms throughout treatment, and whether it is influenced by other variables, such as previous symptom improvements. This issue has been clarified in TA with a therapist. It has been shown that TA with a therapist temporally precedes symptom levels throughout treatment, and that TA with a therapist is not just a by-product of prior symptom improvements (Falkenström et al., 2013; Zilcha-Mano et al., 2014).

## Conclusion

The WAI-VAR has been specifically adapted to measure the TA with ICTs, and it has been shown to have appropriate psychometric properties for its measure. Hence, it is the first validated instrument to be used in clinical and research applications to measure the extent of TA with technologies, in therapies supported by VR and AR. Researching in TA with technologies is an important issue due to it can help us to understand how patients interact with ICTs in therapy, and because of that the WAI-VAR constitutes an excellent instrument that should be used in therapies supported by AR and VR. In addition, the relationship between the WAI-VAR and the WAI-S has been demonstrated. Moreover, this study has revealed the importance of the quality of TA with technologies in achieving CSC in ICT-supported therapies. As Safran and Muran (2000) pointed out, it is important to work directly with the TA, especially if the TA is poor. However, the generalizability of our findings is limited, due to the limitations of the sample. Finally, taking our results into account, and following the changes in the conceptualization by Elvins and Green (2008) and Bordin’s definition of TA (Bordin, 1979), we suggest the conceptualization of WAI-VAR as *“the collaboration between patient and the VR and AR, wherein comfort and trust in the virtual environment and consensus about therapeutic tasks and goals play a central role.*”

## Author Contributions

MM made substantial contribution to the analysis and the interpretation of the data, and drafted the manuscript. RB made substantial contribution to the conception and design of the study, the collection, and interpretation of the data, and revised the manuscript critically for important intellectual content. AC contributed to the conception and design of the study, the collection, analysis, and interpretation of the data, and drafted the manuscript. CB contributed to the conception and design of the study, the collection, and interpretation of the data, and revised the manuscript critically for important intellectual content. All authors provided final approval of the version to be published, and agree to be accountable for all aspects of the work in ensuring that questions related to the accuracy or integrity of any part of the work are appropriately investigated and resolved.

## Conflict of Interest Statement

The authors declare that the research was conducted in the absence of any commercial or financial relationships that could be construed as a potential conflict of interest.

## Abbreviations

AR: Augmented Reality
CSC: clinically significant change
ICTs: Information and Communication Technologies
TA: therapeutic alliance
VR: Virtual Reality
WAI-S: Working Alliance Inventory-Short
WAI-VAR: Working Alliance Inventory applied to Virtual and Augmented Reality.
