# Supplementary material for: Working alliance inventory applied to virtual and augmented reality (WAI-VAR): psychometrics and therapeutic outcomes
Source: Front Psychol. 2015 Oct 8;6:1531. doi: 10.3389/fpsyg.2015.01531 (PMC4597032; doi:10.3389/fpsyg.2015.01531)
Supplement: Supplementary file 1 [file Appendix.DOCX]

**Appendix 1. Working Alliance Inventory applied to Virtual and Augment Reality**

**(WAI-VAR)**

Working Alliance Inventory applied to Virtual and Augment Reality (WAI-VAR)

Please, review today’s session according to these statements. Please circle the score that best describes your opinion, following this system.

| Never | Rarely | Occasionally | Sometimes | Often | Very often | Always |
| --- | --- | --- | --- | --- | --- | --- |
| 1 | 2 | 3 | 4 | 5 | 6 | 7 |

| 1. The virtual environment focuses on the things I have to do to improve my situation. | 1 | 2 | 3 | 4 | 5 | 6 | 7 |
| --- | --- | --- | --- | --- | --- | --- | --- |
| 2. What I am doing in the virtual environment gives me new ways of looking at my problem. | 1 | 2 | 3 | 4 | 5 | 6 | 7 |
| 3. I feel comfortable in the virtual environment. | 1 | 2 | 3 | 4 | 5 | 6 | 7 |
| 4. What I am doing in the virtual environment helps me to accomplish what I want to achieve in therapy. | 1 | 2 | 3 | 4 | 5 | 6 | 7 |
| 5. I trust in the virtual environment’s ability to help me. | 1 | 2 | 3 | 4 | 5 | 6 | 7 |
| 6. The virtual environment works to achieve the therapeutic goals that my therapist and I have agreed on. | 1 | 2 | 3 | 4 | 5 | 6 | 7 |
| 7. I feel received by the virtual environment. | 1 | 2 | 3 | 4 | 5 | 6 | 7 |
| 8. The virtual environment works on the important things that I think I should work on in therapy. | 1 | 2 | 3 | 4 | 5 | 6 | 7 |
| 9. I trust in the virtual environment. | 1 | 2 | 3 | 4 | 5 | 6 | 7 |
| 10. The virtual environment and I have the same ideas about what my problems are. | 1 | 2 | 3 | 4 | 5 | 6 | 7 |
| 11. Thanks to the virtual environment I have achieved a good understanding of the kind of changes that would be good for me. | 1 | 2 | 3 | 4 | 5 | 6 | 7 |
| 12. The way the virtual environment works on my problems is correct. | 1 | 2 | 3 | 4 | 5 | 6 | 7 |
